# Azilsartan as a Potent Antihypertensive Drug with Possible Pleiotropic Cardiometabolic Effects: A Review Study

**DOI:** 10.3389/fphar.2016.00235

**Published:** 2016-08-03

**Authors:** Georgios Georgiopoulos, Vasiliki Katsi, Dimitrios Oikonomou, Georgia Vamvakou, Evangelia Koutli, Aggeliki Laina, Constantinos Tsioufis, Petros Nihoyannopoulos, Dimitrios Tousoulis

**Affiliations:** ^1^1st Department of Cardiology, ‘Hippokration’ Hospital, University of Athens Medical SchoolAthens, Greece; ^2^Department of Internal Medicine, ‘Hippokration’ Hospital, University of Athens Medical SchoolAthens, Greece; ^3^Department of Cardiology, Imperial College London, Hammersmith HospitalLondon, UK

**Keywords:** azilsartan medoxomil, cardiovascular, metabolic, diabetes mellitus, hypertension, pleiotropic

## Abstract

**Background:** Hypertension related cardiovascular (CV) complications could be amplified by the presence of metabolic co-morbidities. Azilsartan medoxomil (AZL-M) is the eighth approved member of angiotensin II receptor blockers (ARBs), a drug class of high priority in the management of hypertensive subjects with diabetes mellitus type II (DMII).

**Methods:** Under this prism, we performed a systematic review of the literature for all relevant articles in order to evaluate the efficacy, safety, and possible clinical role of AZL-M in hypertensive diabetic patients.

**Results:** AZL-M was found to be more effective in terms of reducing indices of blood pressure over alternative ARBs or angiotensin-converting enzyme (ACE) inhibitors with minimal side effects. Preclinical studies have established pleiotropic effects for AZL-M beyond its primary antihypertensive role through differential gene expression, up-regulation of membrane receptors and favorable effect on selective intracellular biochemical and pro-atherosclerotic pathways.

**Conclusion:** Indirect but accumulating evidence from recent literature supports the efficacy and safety of AZL-M among diabetic patients. However, no clinical data exist to date that evince a beneficial role of AZL-M in patients with metabolic disorders on top of its antihypertensive effect. Further clinical studies are warranted to assess the pleiotropic cardiometabolic benefits of AZL-M that are derived from preclinical research.

## Introduction

Hypertension bears an independent association with different indices of cardiovascular (CV) disease, including stroke, myocardial infarction, heart failure, peripheral vascular atherosclerotic disease and end-stage renal disease (Turnbull et al., 2005; Mancia et al., 2014). As clearly stated in the latest European Society of Cardiology guidelines for the management of arterial hypertension, the relationship between blood pressure (BP) and CV morbidity and mortality could be modified by the concomitance of other CV risk factors (Thomas et al., 2001). In fact, metabolic risk factors are more common in hypertensive subjects. Along this line, the prevalence of hypertension and diabetes mellitus are increasing in parallel in industrialized and developing countries, commonly coexist, and patients with both co-morbidities are particularly vulnerable to CV disease and death (Hao et al., 2014). It is not surprising that many patients suffer from CV events despite adequate control of hypertension while on the other hand up to 75% of specific CV complications have been attributed to high BP in diabetic patients (Chen et al., 2011). Under this prism, multiple CV risk factor intervention is required to maximize target-organ protection and should be strongly encouraged where possible (Staessen et al., 2007).

Angiotensin II appears to exert a central role in both the pathophysiology of essential hypertension and arteriosclerosis-associated hypertension (Schmidt-Ott et al., 2000), and insulin resistance (Olivares-Reyes et al., 2009). Therefore, an angiotensin II receptor blocker (ARB) emerges as a reasonable therapeutic strategy of high priority in the management of hypertensive subjects with metabolic co-morbidities. While individual studies reported controversial results regarding the effect of angiotensin-converting enzyme (ACE) inhibitors/ARBs on CV risk in hypertensive patients with type II diabetes mellitus (DMII), a meta-analysis of randomized control studies concluded that this class of drug is associated with significant reduction in CV events and mortality (Hao et al., 2014). Until recently, there were seven ARBs available in the market. Azilsartan medoxomil (AZL-M) is the eighth approved ARB for the management of hypertension (Angeli et al., 2013).

## Objective

Our objective was to evaluate the efficacy, safety, and possible clinical role of AZL-M in hypertensive diabetic patients.

## Methods and materials

A systematic review of the literature for all relevant articles was performed until April 2016 using MEDLINE and COCHRANE LIBRARY. The search strategy implemented the keywords and MeSH terms: “azilsartan,” “azilsartan-medoxomil,” “TAK-491,” “TAK-536,” “Edarbi,” AND “diabetes mellitus,” “type II,” “glucose,” “insulin,” “resistance.” Articles were limited to those published in the English language. A manual search for references from reports of clinical trials or review articles was performed to identify additional relevant studies. Studies were deemed eligible for inclusion when they evaluated in animals (both *in vitro* and *in vivo*), and in human subjects the effect of AZL-M. No further restrictions were imposed on the corresponding recruited population of each study. An additional search in Clinical—Trials.gov (http://www.clinicaltrials.gov) was also conducted to identify recently completed or ongoing trials with AZL-M.

## Results

### Pharmacology

AZL-M is the newest approved ARB for the management of hypertension. It is a prodrug that is quickly hydrolyzed to the active moiety azilsartan and reaches its peak plasma concentration between 1.5 and 3 h following oral administration. It is an ARB with estimated bioavailability of 60% and elimination half-life of 11 h. Metabolism of AZL-M occurs in the liver via cytochrome P450 (CYP) 2C9 and to a lesser extent by CYP2B6 and CYP2C8, resulting in the formation of inactive metabolites. AZL-M is primarily excreted by the kidney, as inactive metabolites, with a clearance of 2.3 mL/min (Angeli et al., 2013). AZL-M is a potent and highly selective and insurmountable AT1 receptor antagonist that binds tightly to and dissociates slowly from AT1 receptors compared with other ARBs (Ojima et al., 2011). In particular, Ojima et al. using indirect kinetic methods showed that AZL-M remained substantially bound to the receptors after washout of the compound compared with other ARBs, such as olmesartan, telmisartan, valsartan, and irbesartan (Ojima et al., 2011) (Supplementary Table 1). As a consequence, the inhibitory effect of AZL-M on AII was reduced only by 25% 4 h after washout while inhibitory effects of olmesartan, telmisartan, and valsartan were reduced by 44, 70, and 99%, respectively (Supplementary Table 1). In the same study, the inhibitory effects of AZL-M on IP1 accumulation and vasoconstriction induced by AII persisted even after washout, whereas those of other ARBs, such as olmesartan and valsartan were markedly attenuated (Supplementary Table 1). In regard to selectivity, AZL-M inhibited the vasoconstriction induced by AII but not vasoconstriction induced by KCl, norepinephrine, 5-hydroxytryptamine, or prostaglandin F2a (Ojima et al., 2011). In addition, the authors reported that AZL-M exhibited inverse agonism against AT1 receptors (Ojima et al., 2011). The high-affinity and tight binding properties of AZL-M are expected to induce potent and long-lasting antihypertensive effects in preclinical and clinical settings while inverse agonism may offer organ protective effects (Ojima et al., 2011). Interestingly, in a small study of hemodialysis patients with cross-over design, AZL-M exerted more prominent suppression of sympathetic nervous system in comparison to other ARBs that resulted in stronger anti-hypertensive effect (Kusuyama et al., 2014). Sympatho-inhibition is a specific class effect of ARBs (Nap et al., 2003), and can be quantified by plasma noradrenaline levels—a sensitive index of the activity of the sympathetic nervous system (Bakris et al., 2000). Thus, the significant decrease in noradrenaline levels following substitution of losartan, valsartan, telmisartan, or olmesartan with AZL-M, suggests an amplified primary class effect for this member of ARBs.

### Preclinical research and metabolic effects of AZL-M

Although AZL-M has been approved for hypertension, it remains to be determined whether this new drug can offer clinical benefits beyond its hypertensive action (Kurtz and Kajiya, 2012). In general, accumulating evidence suggests that ARBs could decelerate the progression of diabetic nephropathy, independently of their BP lowering effect (Weber et al., 2014a; Isaacs and Vincent, 2016). In addition, some ARBs may be more effective than others in reducing proteinuria in patients with diabetic nephropathy despite similar induced reductions in BP (Kurtz and Kajiya, 2012). Most important, specific ARBs are pleiotropic molecules with additional cellular actions beyond the blockade of AT1 receptors that might confer favorable cardiometabolic effects (Kurtz and Pravenec, 2008). According to a relevant review study, ARBs typically have trivial impact on basal glucose and insulin levels, particularly in lean animals, but nonetheless often improve glucose and insulin sensitivity, particularly in obese animals and/or models with type II diabetes (Michel et al., 2016). In addition the authors mentioned that AT1R blockade can improve diabetes-induced vascular remodeling, probably independently of BP lowering (Michel et al., 2016).

Several preclinical studies have shown beneficial effects of AZL-M regarding pro-atherosclerotic pathways, insulin sensitivity, and adipocyte differentiation (Iwai et al., 2007; Kajiya et al., 2011; Kusumoto et al., 2011; Zhao et al., 2011; Lastra et al., 2013; Tarikuz Zaman et al., 2013; Abdelsaid et al., 2014; Matsumoto et al., 2014; Hye Khan et al., 2014a,b; Liu et al., 2016). In specific, Kajiya et al. investigated pleiotropic features of AZL-M in cell-based assay systems independently of its effects on blood pressure and showed that AZL-M, but not valsartan, blocked AII-induced activation of mitogen-activated protein kinases (MAPK) in vascular smooth muscle cells (VSMCs) after delayed washout of the drug (Kajiya et al., 2011). This phenomenon is consistent with the intrinsic high-affinity and tight binding properties of AZL-M (Ojima et al., 2011). However, in the same study AZL-M exerted anti-proliferative effects in vascular cells in the absence of AII, that could be explained by its inverse agonist properties, but also in cells lacking AT1 receptors, suggesting involvement of mechanisms beyond AT1 receptor blockade. Moreover, according to the authors AZL-M impelled a favorable differentiation of adipocytes and exerted stimulatory effects on the expression of genes for PPARa, PPARd, leptin, adipsin, and adiponectin in comparison to a less pronounced effect of valsartan (Kajiya et al., 2011).

On the other hand, animal studies converge that AZL-M is efficient in reducing insulin resistance. Zhao et al. reported that AZL-M improves insulin sensitivity in obese spontaneously hypertensive Koletsky rats and this effect may involve regulation of 11β-HSD1 activity (Zhao et al., 2011). In type II diabetic KK-Ay mice, AZL-M was superior to candesartan in improving glucose intolerance, insulin sensitivity, and inducing beneficial adipocyte differentiation (Iwai et al., 2007). Authors suggested that AZL-M exhibited these effects by reducing TNF-a production and increasing of the expression of PPARγ, C/EBP, and aP2 more effectively than candesartan (Iwai et al., 2007). Toward this direction, an additional study reported that AZL-M improved the *in vitro* insulin effect on glucose transport in red soleus muscle and on the intracellular signaling cascade in the red gastrocnemius muscle in AII-induced insulin-resistant rats (Lastra et al., 2013). The favorable actions of AZL-M in this animal model might be associated with insulin signaling regulation and specifically enhanced AMPKα expression and suppressed p70 S6K1 activation (Lastra et al., 2013).

Recently, AZL-M reduced diabetic kidney damage in Zucker diabetic fatty rats and this effect was accompanied by improved glycemic status, improved vascular homeostasis, reduced BP, and reduced oxidative stress and inflammation (Hye Khan et al., 2014a). In animal models of hypertension and DM with evidence of nephropathy, AZL-M induced superior antihypertensive, insulin-sensitizing and anti-proteinuric effects as compared to olmesartan medoxomil (Kusumoto et al., 2011). Results from another animal study suggest that azilsartan restores endothelial function more effectively than does candesartan cilexetil, by normalizing eNOS function and by reducing inflammation and oxidative stress in diabetic mice (Matsumoto et al., 2014). According to the authors, higher affinity for and slower dissociation from AT1 receptors may underlie AZL-M efficacy in diabetic vascular dysfunction (Matsumoto et al., 2014). Despite consistent favorable effects of AZL-M in nonclinical studies, the metabolic pathways activated by AZL-M in terms of improved insulin sensitivity are not fully elucidated. For example, PPAR-γ is an intracellular receptor involved in the regulation of glucose and lipid metabolism and has gained increasing attention as a novel therapeutic target (Kurtz and Pravenec, 2008). Iwai et al. reported an upregulated expression of PPARγ in adipose tissue (Iwai et al., 2007), but Zhao et al. failed to reproduce this effect (Zhao et al., 2011). In support of the results from Iwai et al., additional data have linked AZL-M with protection of brain endothelial cells from oxidative stress, through preserved mitochondrial function, eNOS mediated anti-inflammatory activity and activation of the PPAR-γ pathway (Liu et al., 2016). Finally, cumulative evidence from other animal studies further strengthens the beneficial cardiovascular effects of AZL-M under settings of unfavorable metabolic profile (i.e., DM or increased insulin resistance, Tarikuz Zaman et al., 2013; Abdelsaid et al., 2014; Hye Khan et al., 2014b). Of note, an observation of clinical significance is that AZL-M in doses similar to those used in humans can improve insulin sensitivity much more than larger doses of other ARBs, such as olmesartan medoxomil or candesartan cilexetil (Iwai et al., 2007; Kusumoto et al., 2011).

### Evidence from clinical trials

#### Anti-hypertensive effects of AZL-M in comparison to other ARBs

A number of double-blind randomized clinical trials have investigated the antihypertensive efficacy and safety of AZL-M compared to other ARBs (Bakris et al., 2011; Sica et al., 2011; White et al., 2011; Rakugi et al., 2012; Table 1). AZL-M was compared with olmesartan in two trials (Bakris et al., 2011; White et al., 2011), with valsartan in two trials (Sica et al., 2011; White et al., 2011), and with candesartan in one trial (Rakugi et al., 2012). The trials ranged in duration from 6 to 24 weeks while the recruited population ranged from 622 to 1291 participants. AZL-M was found to be more effective toward the primary end point of reduction in office or ambulatory systolic BP over each of its comparators. Adverse events were reported similarly in all treatment groups and were mostly mild to moderate in severity, including mainly dizziness, headache, urinary infection, and upper-respiratory tract inflammation. The incidence of treatment-associated adverse events ranged from 35.9 to 65.4% for AZL-M and from 37.9 to 59.2% for alternative ARBs and no significant differences were established for all pairwise comparisons in individual studies (*p* > 0.05 for all; Bakris et al., 2011; Sica et al., 2011; White et al., 2011; Rakugi et al., 2012). Discontinuations due to adverse events were infrequent in all groups. In detail, discontinuation rates for AZL-M ranged from 1.1 to 8.2% as compared with 1.3 to 6.1% for other ARBs (*p* > 0.05 for individual comparisons; Bakris et al., 2011; Sica et al., 2011; White et al., 2011; Rakugi et al., 2012). It should be emphasized that the clinical trials that established the efficacy of AZL-M, analyzed data from 24-h ambulatory blood pressure monitoring (ABPM) for comparisons with alternative antihypertensive agents, in addition to other hemodynamic indices. ABPM provides more reliable predictive data on cardiovascular outcomes than conventional office readings (Fagard et al., 2008). Superiority of AZL-M in lowering BP during a 24 h period could be partially explained by its selective binding and tighter binding properties (Ojima et al., 2011).

**Table 1:** **Clinical trials comparing azilsartan medoxomil as a monotherapy or in specific drug combinations to established antihypertensive medication**.

**Study (year)**	**Type of study**	**Patients**	**Duration**	**Treatment**	**Primary end-point**	**Result**
**AZILSARTAN MEDOXOMIL(AZL-M) AS MONOTHERAPY**						
Bakris et al., 2011	Double–blind RCT	1275	6 weeks	AZL-M $20,40,80 mg or OLM 40 mg or placebo	Change from baseline in 24-h mean SBP	AZL-M 80 mg (−14.6 mmHg) vs. OLM (−12.6 mmHg) (*p* = 0.038) AZL-M 40 mg (−13.4 mmHg) was non-inferior to OLM 40 mg
White et al., 2011	Double–blind RCT	1291	6 weeks	AZL-M 40,80 mg or VAL 320 mg or OLM 40 mg or placebo	Change from baseline in 24-h mean SBP	AZL-M 80 mg (−14.5 mmHg) vs. OLM (–12.0 mmHg) (*p* = 0.009) and VAL (−10.2 mmHg) (*p* < 0.001) AZL-M 40 mg (−13.4 mmHg) was non-inferior to OLM 40 mg
Sica et al., 2011	Double–blind RCT	984	24 weeks	AZL-M 40,80 mg or VAL 320 mg	Change from baseline in 24-h mean SBP	AZL-M 40 mg (−14.9 mmHg) and 80 mg (−15.3 mmHg) vs. VAL (−11.3 mmHg) (*p* < 0.001 for both)
Rakugi et al., 2012	Double–blind RCT	622	16 weeks	AZL-M 20–40 mg or CAND 8–12 mg	Change from baseline in the sitting trough DBP	AZL-M (−12.4 mmHg) vs. CAND (−9.8 mmHg) (*p* = 0.0003)
Bönner et al., 2013	Double–blind RCT	884	24 weeks	AZL-M 20–80 mg or RAM 2.5–10 mg	Change from baseline in the sitting trough SBP	AZL-M 40 mg (−20.6 mmHg) and AZL-M 80 mg (−21.2 mmHg) vs. RAM (–12.2 mmHg) (*p* < 0.001)
Kario and Hoshide, 2015	Open-label RCT^*^	718	8 weeks	AZL-M 20 mg or AML 5 mg	Differences between sleep SBP	AZL-M 20 mg (−12.6 mmHg) vs. AML 5 mg (−17.5 mmHg) (*p* < 0.001)
EARLY Gitt et al., 2016	Observational, prospective^**^	3849	12 months	AZL-M or any ACE-i	Documentation of the achievement of target BP values set according to recent guidelines Description of the safety profile of AZL-M	Target BP level achieved in AZL-M group (61.1% of patients) vs. ACE-I group (56.4% of patients) (*p* < 0.05) Equivalent safety profile (*p* = 0.73)
**AZILSARTAN MEDOXOMIL AS COMBINATION**						
Bakris et al., 2012	Double–blind RCT	609	10 weeks	AZL-M/CLD 40/12.5–40/25 mg or AZL-M/HCTZ 40/12.5–40/25 mg	Change from baseline in clinic SBP	AZL-M/CLD 40/12.5 mg (−35.1 mmHg) vs. AZL-M/HCTZ 40/12.5 (−29.5 mmHg) (*p* < 0.001)
Sica et al., 2012	Double–blind RCT	1714	8 weeks	AZL-M 0,20,40,80 mg and/or CLD 0, 12.5, 25 mg	Change from baseline in trough SBP by ABPM	AZL-M/CLD 40/25 and 80/25 mg (−28.9 mmHg) vs. AZL-M 80 mg (−15.1 mmHg) and CLD 25 mg (−15.9 mmHg) (*p* < 0.001 for both)
Cushman et al., 2012	Double–blind RCT	1071	12 weeks	AZL-M/CLD 40/25 mg or AZL-M/CLD 80/25 mg or OLM/HTCZ 40/25 mg	Changes from baseline in trough, seated, clinic SBP	AZL-M/CLD 40/25 mg (−42.5 mmHg) and AZL-M/CLD 80/25 mg (−44.0 mmHg) vs. OLM/HTCZ 40/25 mg (−37.1 mmHg) (*p* < 0.001)
Weber et al., 2014b	Double–blind RCT	566	6 weeks	AZL-M/AML 40/5,80/5 mg or AML 5 mg + placebo	Change from baseline in 24-h SBP	AZL-M/AML 40/5 mg (−24.8 mmHg) and AZL-M/AML 80/5 mg (−24.5 mmHg) vs. AML 5 mg + placebo (13.6 mmHg) (*p* < 0.001)
Rakugi et al., 2014	Double–blind RCT	603	8 weeks	AZL-M/AML 20/5 mg or AZL-M/AML 20/2.5 mg or AZL 20 mg or AML 5 mg or AML 2.5 mg	Change from baseline in the seated trough DBP	AZL-M/AML 20/5 mg (−35.3 mmHg) and AZL-M/AML 20/2.5 mg (−31.4 mmHg) vs. AZL-M 20 mg (−21.5 mmHg), AML 5 mg (−26.4 mmHg), AML 2.5 mg (−19.3 mmHg) (*p* < 0.001)
Kipnes et al., 2015	Double-blindRCT^***^	299	6 weeks	AZL-M ± CLD ± other antihypertensive or Placebo±CLD ± other antihypertensive (*depending on the open-label phase*)	Change in trough clinic sitting DBP	Mean difference between AZL-M and placebo (−7.8 mmHg) (*p* < 0.001)

* *Multicenter, randomized, open-label, 2-parallel-group study*.

** *Prospective, observational, national, multicenter registry*.

*** *26-week, open-label, titrate-to-target study, followed by a 6-week, double-blind, placebo-controlled reversal phase. Only double-blind reversal phase is reported*.

*RCT, randomized controlled trial; AZL-M, Azilsartan Medoxomil; OLM. olmesartan; RAM, ramipril; VAL, valsartan; CAND, candesartan; ACEi, angiotensin converting enzyme inhibitor; HCTZ, hydroclorothiazide; CLD, clorthalidone; AML, amlodipine; SBP, systolic blood pressure; DBP, diastolic blood pressure; BP, blood pressure; ABPM, ambulatory blood pressure monitoring*.

#### Metabolic effects of AZL-M in comparison to other ARBs

Notable exclusion criteria for most clinical trials assessing AZL-M were previous history of major cardiovascular events or significant cardiac conduction abnormalities, severe renal impairment as well as type I or poorly controlled type II diabetes. As a result, there is limited evidence regarding treatment with AZL-M in diabetic patients. However, three out of the four clinical trials reported the recruitment of patients with well controlled DMII (HbAc1 < 8 mg/dl; Bakris et al., 2011; Sica et al., 2011; White et al., 2011). Although no pre-specified subgroup analysis for the presence of DMII was provided in these studies, we can assume that AZL-M may be effective and well-tolerated in the subgroup of diabetic patients as part of the total population. This assumption is reinforced by the results of the fourth trial by Rakugi et al. (2012). Diabetic patients were included without any limitations and the subgroup analysis based on the presence of DM showed similar effectiveness of AZL-M in the diabetic subgroup compared to the non-diabetic and superiority over candesartan in diabetic subgroup (Rakugi et al., 2012). For all randomized studies of AZL-M vs. alternative ARBs authors did not establish changes in metabolic parameters that could indicate a beneficial metabolic profile of AZL-M in diabetic patients. In a recent meta-analysis of three of the four randomized controlled clinical trials mentioned, the efficacy, safety and metabolic effects of AZL-M were compared to alternative ARBs (valsartan and olmesartan), separately in patients with impaired fasting glucose (prediabetes mellitus) and DM (White et al., 2016). A total of 3821 patients were randomized to either AZL-M, olmesartan, valsartan, or placebo and further stratified by subgroups of normoglycemic, prediabetic, and DM status. AZL-M exhibited more potent antihypertensive efficacy than olmesartan or valsartan in patients with prediabetes mellitus and DM based on both office and ambulatory BP results. As far as metabolic parameters were concerned (including among others blood glucose, insulin levels, and biomarkers, such as adiponectin and lipoproteins), no significant differences were established among treatment subgroups. The authors commented that the short-term nature of the trials incorporated in their pooled analysis may have masked potential beneficial effects of AZL-M on certain metabolic parameters (White et al., 2016).

#### AZL-M as a combination with chlorthalidone

AZL-M was also evaluated for its antihypertensive effect when administered in combination with chlorthalidone (CLD) (Bakris et al., 2012; Cushman et al., 2012; Sica et al., 2012; Table 1). Fixed-dose combinations of AZL/CLD were compared to their individual monotherapies (Sica et al., 2012), to the combination of AZL/hydrochlorothiazide (HCTZ) (Bakris et al., 2012) and with fixed-dose combinations of olmesartan/HCTZ (Cushman et al., 2012). Regarding the primary outcome of reduction from baseline in office systolic BP or 24-h BP, the combination of AZL/CLD was consistently more efficient than its comparators (Bakris et al., 2012; Cushman et al., 2012; Sica et al., 2012). In addition, another study evaluated the efficacy and safety of AZL-M alone and with CLD (Kipnes et al., 2015). Both treatment strategies met the primary endpoint of significant reduction in office change diastolic BP with concomitant long-term stable improvements in BP. The therapeutic effect of AZL/CLD observed in mentioned trials was accompanied by good safety and tolerability (Bakris et al., 2012; Cushman et al., 2012; Sica et al., 2012; Kipnes et al., 2015; Table 1).

#### Anti-hypertensive effects of AZL-M in comparison to other drug classes

For comparisons with different classes of antihypertensive drugs, data on the efficacy, and safety of AZL-M are limited (Bönner et al., 2013; Rakugi et al., 2014; Weber et al., 2014b; Kario and Hoshide, 2015; Gitt et al., 2016; Table 1). As far as comparisons with ACE inhibitors are concerned, a trial evaluated the efficacy and safety of AZL-M vs. ramipril after 24 weeks of treatment. AZL-M was superior to ramipril in reducing all measured indices of BP (trough, clinic, and ambulatory). The safety profile was similar to that of ramipril, with fewer discontinuations due to adverse events (Bönner et al., 2013; Table 1). Albeit not randomized, the one-year outcomes of the EARLY registry, a prospective observational study that was designed to compare AZL-M with ACE-inhibitors under real life settings, further reinforce the results of the aforementioned clinical trials and confirm the greater BP-lowering effect of AZL-M (Gitt et al., 2016; Table 1).

Two randomized trials compared AZL-M in combination with amlodipine with their individual monotherapies (Rakugi et al., 2014), and with amlodipine plus placebo (Weber et al., 2014b), respectively. Changes from baseline in the sitting, trough, diastolic BP and 24 h systolic BP were considered the primary end point and combination of the two drugs was found to be a more efficient regimen than individual monotherapy and placebo along with a similar safety profile (Rakugi et al., 2014; Weber et al., 2014b). In contrast, when AZL-M was compared with amlodipine in a randomized, open-label study (*N* = 718), AZL-M failed to induce a greater reduction in night systolic BP than amlodipine after 8-weeks of treatment (Kario and Hoshide, 2015).

## Discussion

**Figure 1:**
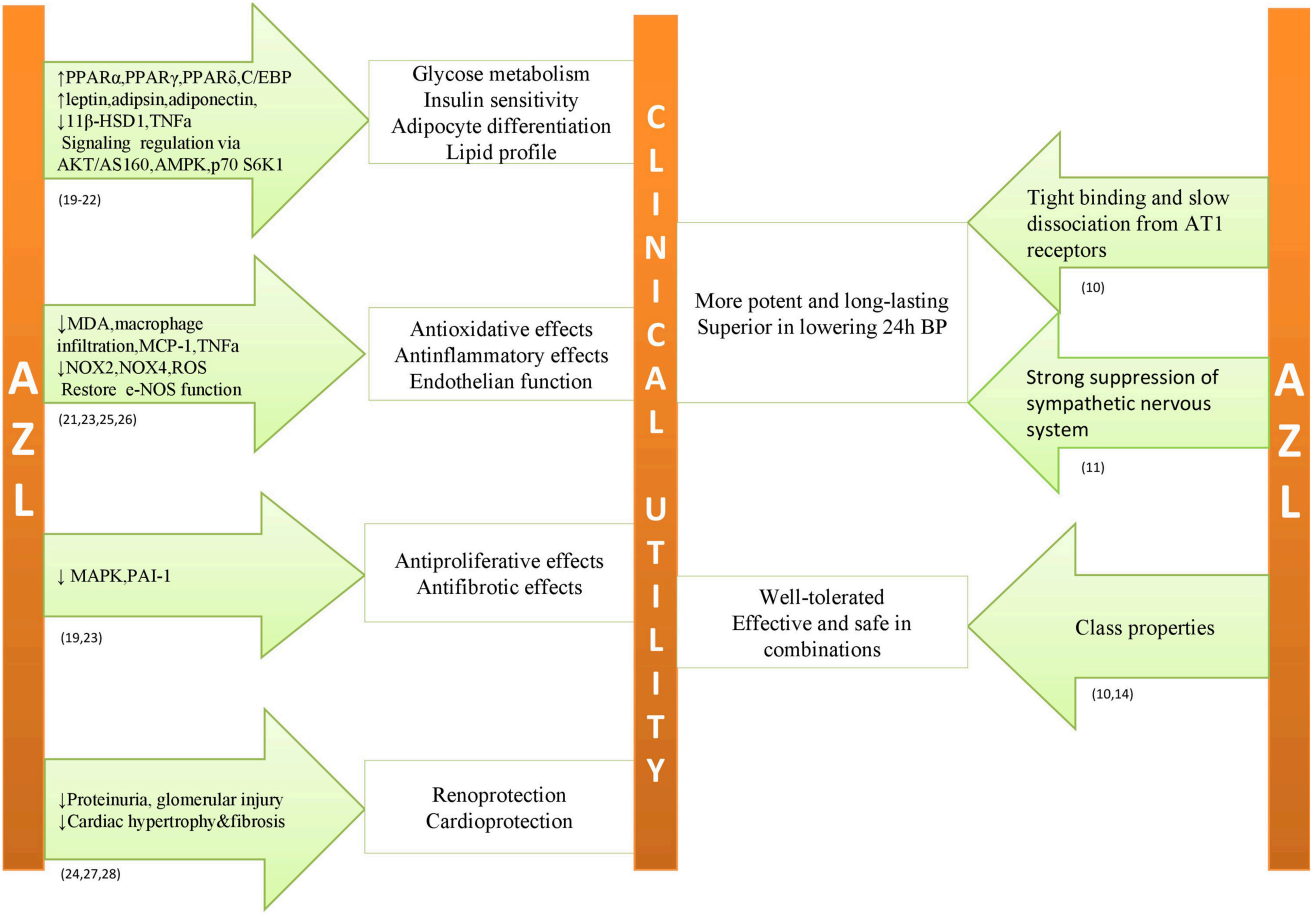
**Possible pleiotropic effects and proposed mechanisms in subjects with unfavorable metabolic profile combined with antihypertensive properties of azilsartan medoximil**.

Based on the preclinical studies (Iwai et al., 2007; Kajiya et al., 2011; Kusumoto et al., 2011; Zhao et al., 2011; Lastra et al., 2013; Tarikuz Zaman et al., 2013; Abdelsaid et al., 2014; Hye Khan et al., 2014a,b; Matsumoto et al., 2014; Liu et al., 2016), AZL-M emerges as a pleiotropic drug that may exert beneficial cardiometabolic effects beyond its antihypertensive properties (Figure 1). These favorable effects are more profound in comparison to other ARBs, probably due to higher affinity, tighter binding, and slower dissociation from AT1 receptors. This superiority was observed even when AZL-M was used in therapeutic doses in comparison to other ARBs that were administered above recommended clinical doses (Iwai et al., 2007; Kusumoto et al., 2011). According to the investigators, the proposed mechanisms of pleiotropic effects are mostly related to AT1 receptor. However, in cellular level there is evidence of actions beyond AT1 receptor (Kajiya et al., 2011). Experimental studies mentioned before converge in the activation of PPARγ pathway from AZL-M as a possible favorable pathophysiological mechanism on top of the AT1 receptor blockage but data is controversial or at least limited in clinical level (Kurtz and Klein, 2009). In general, preclinical trials support AZL-M as a possible beneficial treatment strategy for diabetic patients, beyond its antihypertensive effect. However, no such evidence can be supported from clinical data. Based on the results of up-to date clinical trials (Bakris et al., 2011, 2012; Sica et al., 2011, 2012; White et al., 2011; Cushman et al., 2012; Rakugi et al., 2012, 2014; Bönner et al., 2013; Weber et al., 2014b; Kario and Hoshide, 2015; Kipnes et al., 2015; Gitt et al., 2016), AZL-M is a potent and well-tolerated antihypertensive drug and, furthermore, there is indirect evidence of its efficacy and safety among diabetic patients. In comparison to other ARBs, the superior antihypertensive effect of AZL-M could be attributed to its unique binding properties and, according to a small study (Kusuyama et al., 2014) to the greater suppression of sympathetic nervous system. Moreover, it is efficient when combined with other antihypertensive drugs. At present, there are no large clinical trials assessing the effect of AZL-M on metabolic profile and on cardiovascular morbidity and mortality. Supplementary Table 2 provides a quick reference on the role of AZL-M in the cardiovascular system as assessed by up to date preclinical research and clinical trials.

## Conclusion

In conclusion, AZL-M is an effective and safe BP lowering drug for patients with hypertension and metabolic co-morbidities, including DM or prediabetes mellitus. Pleiotropic cardiometabolic benefits of AZL-M have emerged and validated in preclinical research but should be confirmed and further evaluated in large scale clinical trials. It is now reported on ClinicalTrials.gov that relevant trials have been designed and have proceeded to the recruitment phase. We anticipate primary results from these trials to fully adjudicate AZL-M as the most promising member of the ARB class toward primary and secondary cardiometabolic prevention.

## Author contributions

GG: design of the work, synthesis of data, interpretation of data, final approval. DO, EK, AL: review of the literature, acquisition of data, figure, and table design. VK, CT: conception and design of the work, interpretation of the data. PN, DT: interpretation of data, critical revision, final approval.

### Conflict of interest statement

The authors declare that the research was conducted in the absence of any commercial or financial relationships that could be construed as a potential conflict of interest. The reviewer TG and handling Editor declared their shared affiliation, and the handling Editor states that the process nevertheless met the standards of a fair and objective review.

## Abbreviations

AZL-M: azilsartan medoximil
AII: angiotensin II
AT1: angiotensin II receptor 1
IP1: inositol 1-phosphate
PPAR-γ: Peroxisome proliferator-activated receptor gamma
eNOS: Endothelial Nitric Oxide System.
