# Supplementary material for: Azilsartan as a Potent Antihypertensive Drug with Possible Pleiotropic Cardiometabolic Effects: A Review Study
Source: Front Pharmacol. 2016 Aug 3;7:235. doi: 10.3389/fphar.2016.00235 (PMC4971108; doi:10.3389/fphar.2016.00235)
Supplement: Supplementary file 1 [file Table1.DOC]

**SUPPLEMENTARY MATERIAL**

**AZILSARTAN AS A POTENT ANTIHYPERTENSIVE DRUG WITH POSSIBLE PLEIOTROPIC CARDIOMETABOLIC EFFECTS: A REVIEW STUDY.**

**Authors:** G.Georgiopoulos^1^, V. Katsi^1^, D. Oikonomou^1^, G.Vamvakou^1^, E.Koutli^2^, A.Laina^1^, C.Tsioufis^1^, P.Nihoyannopoulos^1,3^, D.Tousoulis^1^

^1^1st Department of Cardiology, ‘Hippokration’ Hospital, University of Athens Medical School, Athens, Greece

^2^ 1st Department of Internal Medicine, ‘Hippokration’ Hospital, University of Athens Medical School, Athens, Greece

^3^ Department of Cardiology, Imperial College London, Hammersmith Hospital, London, United Kingdom

Supplementary Table 1. Favorable drug specific pharmacological properties of Azilsartan medoximil in comparison to other angiontensin II receptor blockers

| **Inhibitory effects on the specific binding of radiolabeled AII to human**  **AT1 receptors(1)** | | |
| --- | --- | --- |
| **Compound** | IC50(nM) | |
|  | No washout | After washout |
| **Azilsartan** | 2.6 | 7.4 |
| **Olmesartan** | 6.7 | 242.5 |
| **Telmisartan** | 5.1 | 191.6 |
| **Irbesartan** | 15.8 | >10,000 |
| **Valsartan** | 44.9 | >10,000 |
| **Dissociation rate(1)** | | |
| **Compound** | Reduction (%) of inhibitory effects on the specific binding of radiolabeled AII to human  AT1 receptors 240 mins after washout | |
| **Azilsartan** | 25 | |
| **Olmesartan** | 44 | |
| **Telmisartan** | 70 | |
| **Valsartan** | 99 | |
| **Inhibitory effects of on AII-stimulated production of IP1(1)** | | |
| **Compound** | IC50(nM) | |
|  | No washout | After washout |
| **Azilsartan** | 9.2 | 81.3 |
| **Olmesartan** | 12.2 | 908.5 |
| **Valsartan** | 59.8 | 22,664.4 |
| Abbreviations: IC50:half maximal inhibitory concentration; IP1:inositol 1-phosphate,;nM: nanomolar concentration(10^−6^ mol/m^3^); AII: angiotensin II;AT1: angiotensin II receptor 1 | | |

References

1. Ojima et al

| Supplementary Table 2. Key-points on the role of AZL-M in the cardiovascular system as assessed by preclinical research and clinical trials |
| --- |
| - The association between blood pressure (BP) and cardiovascular (CV) morbidity and mortality could be amplified by the concomitance of insulin resistance or diabetes mellitus type II (DMII) |
| - Angiotensin II (AII) receptor blockers (ARBs) are a therapeutic strategy of high priority in the management of hypertensive subjects with metabolic co morbidities |
| - Azilsartan medoxomil (AZL-M) is the eighth approved ARB for the management of hypertension |
| - AZL-M binds tightly to and dissociates slowly from AT1 receptors compared with other ARBs and possibly suppress sympathetic nervous system to a greater extend |
| - In all clinical trials up-to-date, was found to be more effective in terms of reducing indices of BP over alternative ARBs and angiotensin-converting enzyme inhibitors while limited data exist for head to head comparisons with calcium channel blockers |
| - AZL-M is a potent antihypertensive drug when combined with other antihypertensive drugs, especially diuretics |
| - AZL-M is a well-tolerated antihypertensive drug. |
| - Preclinical studies have established pleiotropic effects for AZL-M beyond its primary antihypertensive role |
| - Pleiotropic effects of AZL-M include among others inhibition of mitogen-activated protein kinases (MAPK) in vascular smooth muscle cells, favorable differentiation of adipocytes, differential expression of genes(PPARa, PPARd, leptin, adipsin, and adiponectin) implicated in metabolism, decrease of insulin resistance and upregulation of expression of PPARγ in adipose tissue and anti-inflammatory activity |
| - No clinical data exist that can support a beneficial role of AZL-M in patients with metabolic disorders on top of its antihypertensive effect |
| - Further clinical studies are warranted to assess the pleiotropic cardiometabolic benefits of AZL-M that are derived from animal studies |
